# Underwater Light Manipulation by the Benthic Diatom *Ctenophora pulchella*: From PAR Efficient Collection to UVR Screening

**DOI:** 10.3390/nano11112855

**Published:** 2021-10-26

**Authors:** Edoardo De Tommasi, Ilaria Rea, Maria Antonietta Ferrara, Luca De Stefano, Mario De Stefano, Adil Y. Al-Handal, Marija Stamenković, Angela Wulff

**Affiliations:** 1 National Research Council, Institute of Applied Sciences and Intelligent Systems “E. Caianiello”, Via P. Castellino 111, 80131 Naples, Italy; ilaria.rea@na.isasi.cnr.it (I.R.); antonella.ferrara@na.isasi.cnr.it (M.A.F.); luca.destefano@na.isasi.cnr.it (L.D.S.); 2 Department of Environmental, Biological, and Pharmaceutical Sciences and Technologies, University of Campania “Luigi Vanvitelli”, Via Vivaldi 43, 81100 Caserta, Italy; destefanomario36@gmail.com; 3 Department of Biological and Environmental Sciences, University of Gothenburg, Box 461, 405 30 Göteborg, Sweden; adil.yousif@bioenv.gu.se (A.Y.A.-H.); marie110@gmail.com (M.S.); angela.wulff@bioenv.gu.se (A.W.); 4 Department of Ecology, Institute for Biological Research “Sinisa Stankovic”, University of Belgrade, Bulevar Despota Stefana 142, 11060 Belgrade, Serbia

**Keywords:** biophotonics, diatoms, nanostructured biomaterials, optical properties of pennate diatom frustules, photosynthesis, UVR biological effects

## Abstract

Several species of diatoms, unicellular microalgae which constitute the main component of phytoplankton, are characterized by an impressive photosynthetic efficiency while presenting a noticeable tolerance versus exposure to detrimental UV radiation (UVR). In particular, the growth rate of the araphid diatom *Ctenophora pulchella* is not significantly affected by harsh treatments with UVR, even in absence of detectable, specific UV-absorbing pigments and even if it is not able to avoid high UV exposure by motility. In this work we applied a multi-disciplinary approach involving numerical computation, photonics, and biological parameters in order to investigate the possible role of the frustule, micro- and nano-patterned silica shell which encloses the cell, in the ability of *C. pulchella* to efficiently collect photosynthetic active radiation (PAR) and to simultaneously screen the protoplasm from UVR. The characterization of the photonic properties of the frustule has been accompanied by in vivo experiments conducted in water in order to investigate its function as optical coupler between light and plastids.

## 1. Introduction

Over tens of millions of years, all oceans and freshwaters on our planet have been colonized by diatoms, unicellular microalgae which constitute a major component of phytoplankton [1]. Their efficiency in sunlight harvesting and exploitation is confirmed by the huge contribution that they give to global primary production (estimated to be around 20–25% [2]). Their impact on Earth biogeochemistry is also related to their ability to synthesize nanostructured silica shells, the frustules, starting from monosilicic acid (Si(OH)_4_) dissolved in water [3], leading to about 240 Tmol of annual biogenic silica precipitation [4]. The extreme diversity in frustule morphology is at the basis of their taxonomy, with more than $10^{3}$ genera identified so far and about $10^{5}$ estimated species [5]. Despite this variability, there are some characteristics that are common to all genera. Most frustules (with dimensions ranging from few microns up to 1 mm) have a “petri-dish like” shape [6], with an *epitheca* overlapping a *hypotheca*, every theca being constituted by a valve and one or more lateral bands (the girdles) [7]. Valves and most girdles are perforated by regular, periodic patterns of pores, whose dimensions (with diameters ranging from tens of nanometers to about 1 micron) and periodicity are species-specific and also depend on the position they occupy in the frustule [8]. Valves may be multi-layered, every layer being characterized by different pore dimensions and lattice constants [9]. Frustules symmetry allows distinguishing between *centric* diatoms (mainly planktonic and characterized by round or polygonal valves) and *pennate* diatoms (commonly benthic and provided with bipolar, elongated valves) [7].

Possible functions of the frustule include mechanical protection, being able to resist pressures up to 7 N ${\mathrm{mm}}^{-2}$ (equivalent to 700 t $m^{-2}$) [10]; filtering of silicic acid and nutrients (mainly ${\mathrm{NH}}_{4}^{+}$, ${\mathrm{HCO}}_{3}^{-}$, and ${\mathrm{NO}}_{3}^{-}$) from noxious agents (e.g., viruses) and gas exchange [11,12]; sinking speed lowering (for planktonic species) if compared to equivalent spherical particles, implying a longer stay of the cell near water body surface and a consequent longer exposition to sunlight [13]; efficient coupling with light [14], which could explain the extraordinary photosynthetic productivity of diatoms. In particular, it has been observed the ability of valves of several centric species to collect and confine photosynthetic active radiation (PAR) in intense hot-spots [15,16,17], and, in several cases, both valves and girdles have shown to behave like photonic crystal slabs [18,19]. Since the XIX century up to recent days, physical and chemical properties of the frustule have been exploited in several fields [20], comprising microscope objectives quality testing [7], water filtration [21], forensic science [22], optics and photonics [19,23], plasmonics [24,25], catalysis [26], biochemical sensing [27,28], solar energy harvesting [29,30], biomedicine and drug delivery [31,32].

In recent years, the possible role of diatom frustule in screening from detrimental ultraviolet radiation (UVR) has been scrutinized [33,34,35,36], even though this property has never been studied in conjunction with experiments conducted on living cells in their aquatic environment, but only by characterizing the optical properties of the bare shells in air. For wavelengths below 200 nm, UVR is strongly absorbed by atmospheric nitrogen and oxygen. UV-C radiation (λ= 100–280 nm) is efficiently absorbed by stratospheric ozone, however part of UV-B radiation (λ= 280–315 nm) is able to reach Earth surface and significant depths (up to 60–70 m) in clear coastal and oceanic waters [37,38]. Main damages induced by exposure to UV-B radiation are related to the formation of dimeric photoproducts between adjacent pyrimidines in DNA [39], which in turn may induce, in phytoplanktonic microalgae, inhibition of cell division and photosynthesis, decrease in nutrient uptake and protein synthesis, and loss of photo-orientation and motility [37,40]. For sufficiently high intensities, UV-B may even lead to cell death [39]. Even though most microalgae are able to rely on several mechanisms of protection versus UV-B exposure (e.g., photo-enzymatic repair photoreactivation (PER), synthesis of photoprotective pigments such as carotenoids and mycosporine-like amino acids, MAAs), a great variability in UVR susceptibility has been observed across microalgal genera and even species [37,38,41]. In particular, diatoms are characterized by low levels of UV-absorbing compounds, especially MAAs, if compared to dinoflagellates, cryptophytes, prymnesiophytes, and raphidophytes, and in some cases they are not detectable at all even by high performance liquid chromatography (HPLC) [40,42]. Nevertheless, several diatom species are characterized by a noticeable tolerance to UV-B, with poor effects on growth rates [38] and low induction of DNA lesions [37,43]. UVR tolerance has in particularly been observed for benthic pennate species in both laboratory [44] and field experiments [42,45,46] also when exposed to UV-B and UV-A intensities/doses exceeding ambient radiation conditions [43,44].

When exposed to high light levels, motile benthic diatoms, usually inhabiting sediment and microbial mats [37], are able to migrate downwards, as observed for example for *Gyrosigma balticum* [47,48], thus limiting detrimental effects induced by UVR. This is not the case for *Ctenophora pulchella*, a cosmopolitan araphid (non-motile) pennate diatom which is still characterized by high resistance versus both UV-A and UV-B irradiation, even if motility is precluded and the presence of UVR-absorbing compounds is scarcely detectable.

The aim of this work is to understand if and at which extent *C. pulchella* frustule is able to protect the cell from UVR exposure, and in general its role in light manipulation, including PAR efficient collection. Nevertheless, the present study is not limited to the analysis of bare frustules as in previous literature on this topic. A complete numerical and experimental characterization of frustule optical properties has been indeed followed by observations of their effects on living individuals immersed in water, which represents, to the best of our knowledge, an approach that has never been followed so far.

## 2. Materials and Methods

### 2.1. Sampling, Culture Conditions, Frustule Cleaning and Microscopy

*C. pulchella* cells were obtained as epiphytes on several brown macrophytes collected from Västervik, on the Baltic coast of Sweden (57°44′21 N 16 40 04). After isolation, cells were kept in 50 mL Nunc flasks filled with F/2 medium in a climate chamber at 16 °C and 40 μmol photons $m^{-2}$ $s^{-1}$ (16 h light and 8 h darkness). The light was provided by white fluorescent tubes (Philips Master, TL-D 58W/840, Reflex Eco, Amsterdam, The Netherlands) and was adjusted using a cosine quantum sensor (LI-COR, LI-1400, Lincoln, NE, USA). For frustule cleaning, the samples were boiled in 35% hydrogen peroxide for 15 min to remove all organic material in the frustule. Clean frustules were then rinsed several times in deionized water to remove salt residuals.

For light microscopy, few drops of the cleaned material were left to dry on a cover slip in ambient temperature and then mounted in Naphrax, a synthetic mountant with high refractive index. Morphometric measurements of the frustules and imaging were made under Zeiss Axioimager 2 microscope with differential interference contrast (DIC) objectives and Canon Powershot 14 digital camera (Department of Biological and Environmental Sciences, University of Gothenburg, Sweden).

For scanning electron microscopy (SEM) observations, the cleaned diatom aliquot was filtered on 1 μm Nucleopore Whatman filter and left to dry on an aluminum stub covered with conductive and adhesive black carbon disks before coating with gold palladium alloy. A LEO Ultra 55 SEM (Chalmers University, Gothenburg, Sweden) was used for examining and imaging. Examination was made on 8 kV at a distance of 8 mm.

To characterize plastids, cells from different treatments were examined using confocal microscopy at Chalmers University, Gothenburg, Sweden (Nikon Ti-E with A1-DUG DU-Hybrid Detector Unit with PMT and A1 Filter Cube 595/50 700/75). Nikon LU-N4 laser box: Spectral Physics 640 nm. CFI Apo λS 60XOil objective with nano-crystal coat. 3D-image stacks that were recorded were visualized as 2D maximum intensity projections.

### 2.2. UVR Treatments

To test the influence of PAR and UVR on physiological parameters, cells of *C. pulchella* were taken from the middle of logarithmic phase and transferred in 25 mL quartz flasks to the starting cell number of $1.3\times 10^{7}$ cells $L^{-1}$, with the addition of 10 mL fresh medium (n=3). Background PAR was provided by the same white fluorescent tubes, while UV radiation was provided by UV lamps (Q-Panel UV-A-340, 40 W, Cleveland, OH, USA), emitting a spectrum qualitatively similar to solar radiation in the range of 290 to 340 nm. To cut different wavebands off the whole spectra emitted by the fluorescent tubes, quartz bottles were covered with one of the following filters: Ultraphan transparent (280–700 nm; Digefra GmbH, Munich, Germany), Folanorm (320–700 nm; FolexGmbH, Dreieich, Germany), and UltraphanURUV Farblos (400–700 nm; Digefra GmbH, Munich, Germany) corresponding to the PAR + UV-A + UV-B (PAB), PAR + UV-A (PA), and PAR treatments, respectively. These commercially available filters’ cutoff wavelengths slightly differed from the UVR definition of CIE (Commission Internationale De l’Eclairage, UV-B = 280–315 nm, UV-A = 315–400 nm [49]). UVR was measured using a Solar Light PMA2100 radiometer equipped with the UV-A sensor PMA 2110 and the UV-B Sensor PMA 2106 (Solar Light, Philadelphia, PA, USA). Adjusted UVR below the cutoff filters was 5 W $m^{-2}$ for UV-A and 1–1.4 W $m^{-2}$ for UV-B (13 h irradiation), while the experimental PAR was adjusted to 120 μmol photons $m^{-2}$ $s^{-1}$ (16 h irradiation), at 16 °C. The PAR, PA and PAB treatments lasted 7 days, and samples for the estimation of photosynthetic activity and growth rates were taken at the start and at the end of cultivation. After the treatments, cultures were exposed to low PAR (40 μmol photons $m^{-2}$ $S^{-1}$) for 24 h recovery. Another set of flasks with *C. pulchella* cells was placed in a climate chamber under standard growth conditions (40 μmol photons $m^{-2}$ $s^{-1}$, 16 h light, 16 °C) and it was designated as a control (n=3).

### 2.3. Growth Rate

The cell number was estimated using a gridded Sedgewick Rafter counting chamber under a light microscope (Zeiss, Axiovert 40, Oberkochen, Germany), and specific growth rate per day (μ) was calculated by the formula:

(1) $$\mu =\frac{\ln\left(\frac{N_{1}}{N_{0}}\right)}{t_{1}-t_{0}}$$

where $N_{1}$ and $N_{0}$ are the cell concentrations at the end and beginning of a period of time *t* days.

### 2.4. Chlorophyll Fluorescence Measurements

Photosynthetic efficiency was measured as the fluorescence of PSII, determined by using a pulse amplitude modulation fluorometer (Water PAM, Walz GmbH, Effeltrich, Germany) connected to a computer with WinControl software (Walz GmbH, Effeltrich, Germany). Prior to measurement, the number of cells was equalized by adding a quantity of medium to achieve approximately 7700 cells ${\mathrm{mL}}^{-1}$. Immediately after sampling, the algal suspension was acclimated in darkness for 5 min at 16 °C and put in 5 mL Quartz cuvettes (Hellma, Müllheim, Germany). The suspensions were gently stirred using a small magnetic bean during the fluorescence measurements. The maximum quantum yield ($F_{v}/F_{m}$; the ratio of variable to maximum chlorophyll fluorescence from photosystem II) was measured at time zero (n=6). After dark incubation, a pulse of weak, far-red light was applied to empty the electron pool from $Q_{A}$. The initial fluorescence ($F_{0}$) was measured with red measuring light (~0.3 μmol photons $m^{-2}$ $s^{-1}$, 650 nm) and the maximum fluorescence ($F_{m}$) was determined using a 600 ms completely saturating white light pulse (~3500 μmol photons $m^{-2}$ $s^{-1}$).

Photosynthesis (in terms of the relative electron transport rate, $rETR=PFR\cdot \frac{\Delta F}{F_{m}^{\prime }}$) versus irradiance curves were also measured (rETR curves, n=3). Here, PFR refers to photon fluence rate; $F_{m}^{\prime }$ is the maximum fluorescence from a light adapted sample; ΔF (or $F_{q}^{\prime }$) refers to the difference in fluorescence between $F_{m}^{\prime }$ and $F^{\prime }$; $F^{\prime }$ is the fluorescence emission from an irradiated sample [50]. Thirteen levels of light intensity from white light LED of the Water PAM, ranging from 5 to 1679 μmol photons $m^{-2}$ $s^{-1}$, were used to create the rETR curves, the duration of each intensity being 30 s. The hyperbolic tangent model of Jassby and Platt (1976) was used to estimate rETR curve parameters described as:

(2) $$rETR=rETR_{max}\cdot \tanh\left(\alpha \cdot PFR\cdot rETR_{max}^{-1}\right)$$

where $rETR_{max}$ is the maximum relative electron transport rate, tanh is the hyperbolic tangent function and α is the electron transport efficiency. The saturation irradiance for electron transport ($I_{k}$) was calculated as the light intensity at which the initial slope of the curve (α) intercepts the horizontal asymptote ($rETR_{max}$—the maximum relative electron transport rate). The curve fit was calculated with the Solver Module of MS-Excel using the least squares method and comparing differences between measured and calculated data. Along with $F_{v}/F_{m}$, the parameters of rETR curves: $rETR_{max}$ which determines the photosynthetic capacity, the slope of rETR curve (α) referring to the photosynthetic efficiency, and the saturating irradiance ($I_{k}$) are considered essential in the assessment of UVR-stress influences on the physiological state of PSII [51,52].

### 2.5. Numerical Simulations

Light propagation through single valves and inside whole cells of *C. pulchella* have been performed at different wavelengths and in different environmental conditions making use of the Wide-Angle Beam Propagation Method (WA-BPM), which represents, as the name suggests, a generalization of BPM to non-paraxial conditions (as it is in the case of light transmitted and diffracted by the frustule ultrastructure) based on multi-steps Padé wide-angle technique [53,54]. Simulations have been carried out by BeamPROP engine in the RSoft CAD environment (Synopsis) and run on a DELL workstation (Xeon bi-processor with 2 × 14 cores, 512 GB RAM, graphic board NVIDIA QUADRO K6000). 3D CAD models of the frustule have been retrieved by SEM images of the inner and outer sides of a single valve. The gray-scale SEM images have been converted into binary images after applying a proper threshold. Binary images have been in turn used to derive refractive index maps of both valves and surrounding environment. Extrusion of valve refractive index maps allowed the construction of 3D CADs. This methodology represents an improvement compared to ab-initio CAD designs usually reported in literature (where frustule ultrastructure is characterized by perfect periodicity), since SEM morphology characterization allows an accurate reproduction of valve imperfections and irregularities. Silica dispersion and absorption have been taken into account. Deatils on WA-BPM algorithm, frustule CAD retrieval and whole cell CAD design are reported in Supplementary Materials.

### 2.6. Transmission Imaging

Spatial distribution of light intensity transmitted by individual valves in different regions of the optical spectrum has been retrieved by the following experimental set-up. A UV-VIS lamp (Hamamatsu Photonics, Hamamatsu, Japan, model L10290) provided with optical fiber output has been used as source of partially coherent radiation; it includes a deuterium lamp (with emission between λ=200 nm and λ=400 nm) and a tungsten halogen lamp (with emission in λ = 400–1100 nm interval). The two lamps can be used independently or simultaneously. Furthermore, optical band-pass filters allowed working in narrower spectral windows: 280–315 nm (Asahi Spectra, Torrance, CA, USA, UV-B filter), 460 nm (Thorlabs, Newton, NJ, USA, FL460-10, 10 nm FWHM), 532 nm (Thorlabs, FL532-10, 10 nm FWHM), and 640 nm (Thorlabs, FB640-10, 10 nm FWHM). Radiation is emitted through an optical fiber transparent in 200–700 nm spectral range (Hamamatsu, A7969 anti-solarization fiber) provided with a quartz collimator (Lot Oriel, LLZ010). Sparse valves were deposited onto a fused quartz microscope slide (UQG optics, FQM-7521, transmission >90% between 280 nm and 2 μm) and invested by incoming radiation. Single valves were selected by means of a micrometric xyz translational stage (Thorlabs, PT3/M). The transmitted light was collected by a microscope objective (Zeiss, Oberkochen, Germany, 50X Epiplan, NA 0.7 for measurements in visible spectral range; Thorlabs, LMU-40X-UV-B, NA 0.49, AR coatings in 240–360 nm range for measurements in UV-B). The objective was mounted on a micrometric translation stage (Thorlabs, PT3A/M) and images have been acquired with a 1 μm step. A CMOS camera (IDS, UI-1240SE-C-HQ, quantum efficiency: >40% in red, green, and blue spectral windows) has been used for measurements in visible spectral range and a UV-sensitive CCD camera (Hamamatsu, C8484-16C, quantum efficiency: 25–33% for λ = 280–315 nm) for measurements in UV-B. In order to avoid to detect undesired visible photoluminescence induced by UV excitation, a further UV-B bandpass filter has been inserted in the inlet of the CCD camera. The acquired images were analyzed and compared with light transmitted by a portion of the quartz slide without any valve. For measurements with living *C. pulchella* cells, a water suspension of microalgae has been injected into a chamber (25 × 25 mm wide, ~50 μm thick) obtained by sealing with silicone the edges of two UV fused silica coverslips (UQG optics, CFS-2525, transmission >90% between 175 nm and 1.2 μm).

### 2.7. Digital Holography

#### 2.7.1. Hologram Acquisition

Digital Holographic Microscopy (DHM) is a powerful imaging technique giving the possibility to quantitatively reconstruct objects in 3D. Holograms are particular images acquired by using a laser source with high spatial and temporal coherence in an interferometric configuration. In particular, in this study, a linearly polarized laser source emitting at λ=660 nm, with an output maximum power of 700 mW in continuous wave (CW) and a coherence length ≃100 m, was used. This optical beam was collected and splitted through an objective lens (10X, NA 0.22) coupled with an optical fiber coupler (Single Mode Fiber Coupler, 1 × 2, 70/30, FC/APC); the two emerging beams are known as object and reference beams. To improve the fringe contrast, a λ/2 wave plate was positioned in the object beam. The object beam was collected by using a microscope objective lens with a magnification of 63X (NA 0.75). Then, the reference and the object beams were recombined by means of a beamsplitter and the resulting interference pattern has been collected onto the surface of a CCD camera (1024 × 768 pixel array; pixel size Δx=Δy=4.65 μm). In order to compensate all the aberrations introduced by the optical components, including the defocusing due to the microscope objective, a double exposure technique was adopted: one acquisition was made on the sample under investigation, while the second one was made on a reference surface in proximity of the object. Aberrations can be compensated by numerically manipulating the two holograms [17].

#### 2.7.2. Amplitude and Phase Reconstruction

The amplitude and phase maps of the object under test can be mathematically retrieved by applying an image reconstruction procedure. Briefly, starting from the frequency spectrum of the acquired hologram in off-axis configuration, the first diffraction order is separated from the whole spatial frequency spectrum with a bandwidth filter and shifted to the origin of the plane. As a result, the spectrum of the object field (defined as $O\left(x,y\right)=\left|O\left(x,y\right)\right|e^{i\phi (x,y)}$, with |O(x,y)| and ϕ(x,y) amplitude and phase, respectively, and *x* and *y* cartesian coordinates defining the plane of acquisition of the hologram) is obtained except for a constant [55]. The optical wavefront at different distances from the plane of acquisition can be also reconstructed starting by the retrieved whole object field and applying the Fourier formulation of the Fresnel-Kirchhoff diffraction formula [56]. A different approach to the propagation of the field is based on the replacement of the Fresnel-Kirchhoff integral, the lens transfer factor, and other operations by operator algebra in the Fresnel diffraction description [57]. As a consequence, the description of Fourier optics appears more simple and compact. In particular, the propagated field $O_{prop}\left(\xi ,\eta \right)$ as a function of the initial field O(x,y) can be expressed as [17]:

(3) $$O_{prop}\left(\xi ,\eta \right)=\exp\left(ikd\right)\times \left\{F^{-1}\left[\exp\left(-\frac{ikd\lambda^{2}}{2}\left(\nu^{2}+\mu^{2}\right)\right)\right]\cdot F\left(O(x,y)\right)\right\}$$

here F[f(x)] is the Fourier transform of the function f(x), $k=\frac{2\pi n}{\lambda }$ (with *n* refractive index of the medium), ν and μ are spatial frequencies defined as $\nu =\frac{\xi }{\lambda d}$ and $\mu =\frac{\eta }{\lambda d}$, and *d* is the reconstruction distance. For digital reconstruction Equation (3) is implemented in a discrete form:

(4) $$O_{prop}\left(m,n\right)=\exp\left(ikd\right)\left\{F_{D}^{-1}\left[-\frac{ikd\lambda^{2}}{2N^{2}d^{2}}\left(U^{2}+V^{2}\right)\right]\cdot F_{D}\left(O\left(h,j\right)\right)\right\}$$

where *N* is the number of pixels in both directions and *m*, *n*, *U*, *V*, *h* and *j* are integer numbers varying from 0 to N−1. The discretized Fourier transform is defined as:

(5) $$F_{D}\left\{g\left(j,l\right)\right\}=\frac{1}{N}\sum_{j,l=0}^{N-1}\exp\left[-\frac{2\pi i}{N}\left(mj+nl\right)\right]g\left(j,l\right)$$

Intensity and phase distributions of the propagated field can be evaluated by using the following relations:

(6) $$\begin{array}{c} I_{prop}\left(m,n\right)={\left|O_{prop}\left(m,n\right)\right|}^{2} \end{array}$$

(7) $$\begin{array}{c} \phi_{prop}\left(m,n\right)=\arctan\frac{Im[O_{prop}\left(m,n\right)]}{Re[O_{prop}\left(m,n\right)]} \end{array}$$

As can be noticed in Equation (7), the reconstructed phase distribution is obtained by a numerical evaluation of the arctan function, thus its values are restricted in the interval [−π,π], i.e., the phase distribution is wrapped into this range. To avoid possible ambiguities due to thickness differences greater than λ/2, phase-unwrapping methods have to be generally applied [17].

### 2.8. Fluorescence Imaging and Photoluminescence Spectroscopy

Diatom valves of *C. pulchella* were deposited on a silicon wafer and their emission properties investigated by both fluorescence imaging microscopy and steady-state photoluminescence (PL) analysis. The fluorescence images of a single valve were acquired by using a Leica DM6M microscope (Leica Microsystems, Wetzlar, Germany) equipped by the solid-state fluorescence light source Leica SFL4000, and controlled via LAS X (Leica Application Suite; rel. 3.0.13) software. The imaging was performed with a 50X objective in dry medium, exciting the fluorescence emission by two LED modules at 365 and 470 nm. The steady-state PL of the valves was excited by a continuous wave He-Cd laser (KIMMON Laser System) with emission wavelengths at 325 and 442 nm. PL emission was collected at normal incidence to the surface of the sample through an optical fiber, then dispersed by a spectrometer (Princeton Instruments, Trenton, NJ, USA, SpectraPro 300i), and detected using a Peltier cooled charge coupled device (CCD) camera (PIXIS 100 F). Long pass filters with a nominal cut-on wavelength of 350 and 458 nm were used to remove the laser line at monochromator inlet for excitation at 325 and 442 nm, respectively.

## 3. Results and Discussion

### 3.1. Morphological Characterization of the Frustule

**Figure 1:**
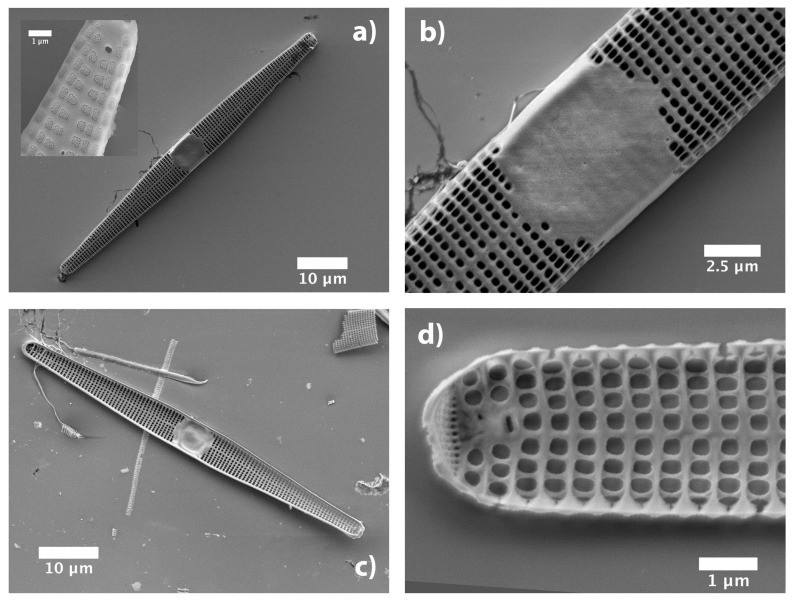
SEM images of a single valve of a *C. pulchella* diatom. (**a**) External view and detail where intact *cribrum* is visible (inset). (**b**) Central *fascia*. (**c**) Internal view. (**d**) Detail of the inner side.

*C. pulchella* is a benthic, araphid (non-motile) diatom which is usually found attached to substrata by mucilage. It is widely distributed both in brackish and fresh water [7]. In our natural population samples, the valve (45–90 μm long, 3–3.5 μm wide) is linear to linear-lanceolate with rounded apices. This feature might be changed in culture strains and growth stage where valve width may reach 6–8 μm (own observation). Transverse, uniseriate *striae* are composed of elliptical or quasi-squared *areolae* (pores) with linear dimensions of 250–300 nm (thus comparable with optical wavelengths). The pores are closed externally by *cribrae* (see inset in Figure 1a), ornate by complex patterns of holes with a mean diameter of about 30 nm. Apical pore patterns are also distinguishable (see the tip of the valve in Figure 1d). The valve center is characterized by the presence of a broad plain area (nodule or *fascia*, see Figure 1b), with a very narrow longitudinal *sternum* (Figure 1d). *Fascia* may have ghost striae. *Valvocopula* is non-areolate but other bands may have a single row of pores (not shown). The height of the whole frustule is of about 10 μm. SEM characterizations have been performed on n=6 individuals.

### 3.2. Light Propagation through a Single Valve

**Figure 2:**
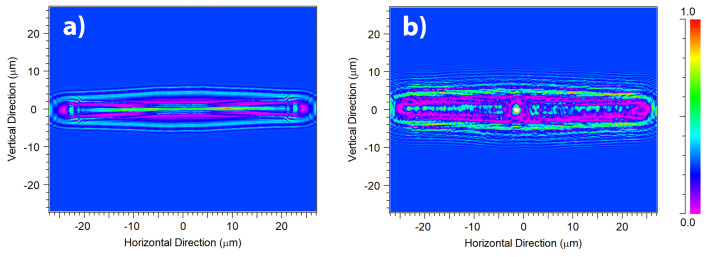
Transmitted intensity map in XY plane numerically evaluated at a distance z=13 μm from the valve when immersed in air and illuminated by a plane wave at λ=633 nm. The map in (**a**) refers to an imaginary valve not provided with pores and central *fascia*, while the map in (**b**) refers to a real valve. Incoming intensity: 0.25 (a.u.).

**Figure 3:**
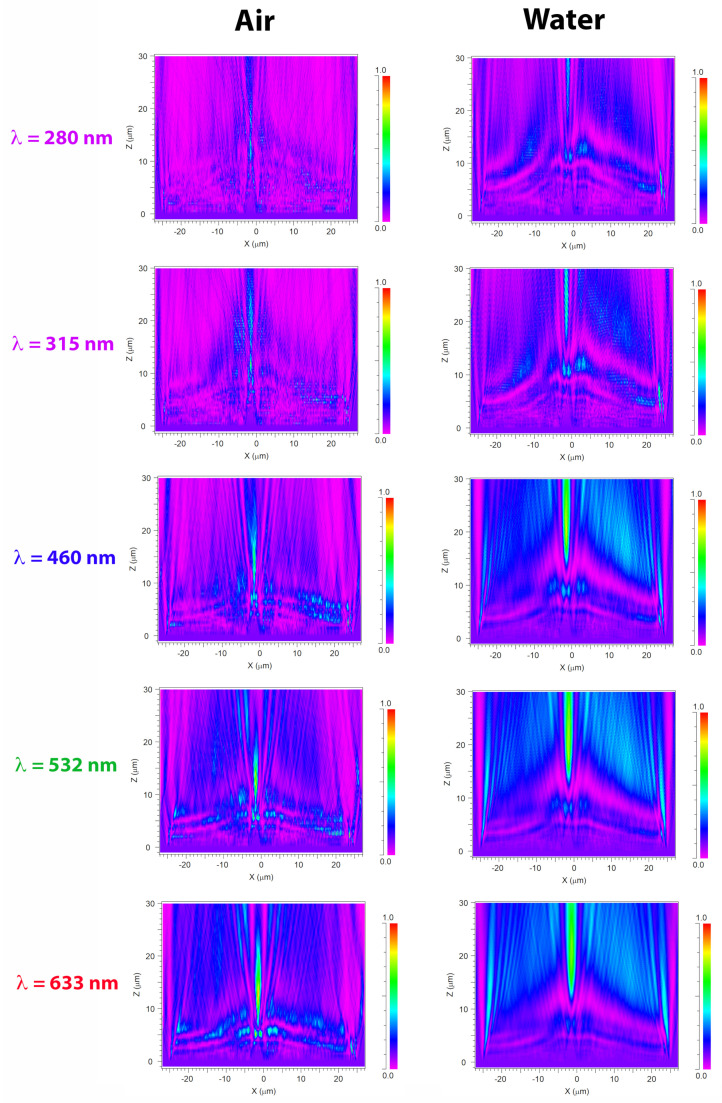
Numerical simulations referring to the intensity of transmitted radiation in XZ plane, *z* being the direction of propagation of light. The valve lies in XY plane between z=0 and z=0.4 μm. Simulations have been performed both in air (left column) and in water (right column) and for different wavelengths in UV-VIS spectral range (see rows). Incident power: 0.1 (a.u.)

A crucial aspect in the interaction between light and diatom frustules is the role of their ultrastructure. In this perspective, it can be useful to look at the optical behavior of a silica plate with the same shape and dimensions of a valve, but lacking of pores and *fascia* (see the results of numerical simulations reported in Figure 2a and performed in air for an incoming plane wave with wavelength λ=633 nm). This imaginary valve behaves like a cylindrical lens, confining light only along one direction in virtue of refraction. The effect of diffraction from the boundaries of the plate is also visible. When considering a real valve provided with its ultrastructure (see Figure 2b), multiple diffraction contributions coming from the pores constructively interfere with each other and add to the light refracted by the *fascia* (which, being a non-porous silica plate, in fact acts like a microlens), thus the confinement of radiation takes place both along the apical direction (transversal confinement) and along the direction of propagation of light (longitudinal confinement along the optical axis z) with the formation of an intense hotspot. The overall resulting effect is an efficient collection of radiation in multiple directions. It is worth noticing that the presence of *cribra* has not been taken into account in the simulations because in this case the linear dimension of the pores is one order of magnitude smaller than the optical wavelengths, thus they do not induce detectable diffraction when hit by light [58]. The divergence of radiation caused by pores diffraction is more evident if we look at the transmitted intensity in XZ plane (or, equivalently, in YZ plane), i.e., the plane of light propagation (the XY plane being the one in which the valve lies), as can be seen in Figure 3.

In this case the simulations have been performed for a valve immersed in air (left column) and in water (right column) for different wavelengths of the incoming radiation. In particular, the extremes of UV-B spectral range (λ=280 and λ=315 nm) and the main components of PAR (blue radiation at 460 nm, green radiation at 532 nm, and red radiation at 633 nm) have been considered, taking into account silica dispersion and absorption [59] and water dispersion (water absorption being negligible up to near infrared) [60]. The valve extends from z=0 to z=0.4 μm and the transmitted intensity is evaluated up to z=30 μm. In UV-B spectral range, incident radiation undergoes significant absorption by frustule silica. Furthermore, the shorter the wavelength, the lower the divergence angle of the light diffracted by the pores and generally by the ultrastructure of the frustule, thus for UV-B radiation the region of coherent superposition of the diffraction contributions is less extended compared to PAR radiation and takes place at a greater distance from the valve. We can conclude that the absorption and diffraction properties of the frustule are such that the incident radiation is more efficiently collected and confined at longer wavelengths (PAR) compared to UVR. This trend is present also when the valve is immersed in water, even though the lower refractive index contrast between the silica and the surrounding environment causes a slight shift in the position of the light confinement region towards longer distances along the optical axis. It can be also noticed how the light confined along z is less intense but more widespread compared to propagation in air.

**Figure 4:**
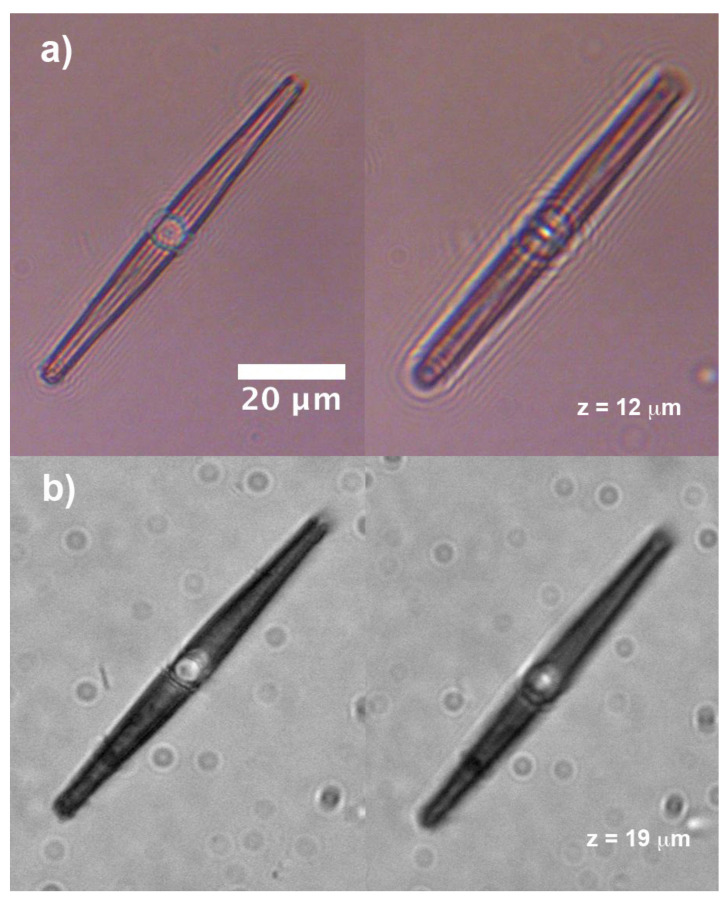
Image of a single valve of *C. pulchella* illuminated by VIS-NIR radiation (λ = 400–1100 nm) and acquired at z=0 (left) and z=12 μm (right) along the optical axis (**a**). Image of the same valve illuminated by UV-B radiation (λ = 280–315 nm) and acquired at z=0 (**left**) and z=19 μm (**right**) along the optical axis (**b**).

**Figure 5:**
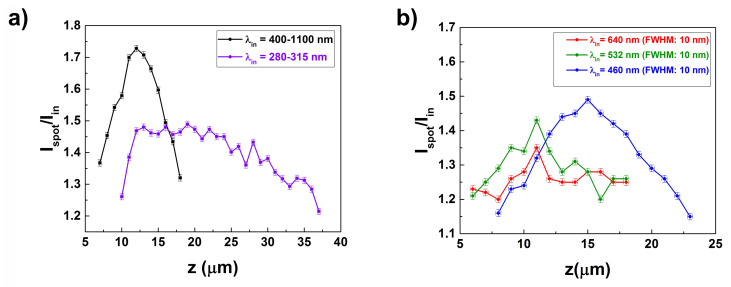
Ratio of the hotspot intensity ($I_{\mathrm{spot}}$) to the incident intensity ($I_{\mathrm{in}}$) as a function of the distance *z* from the valve and for different spectral ranges: VIS-NIR [black curve in (**a**)]; UV-B [violet curve in (**a**)]; red, green, and blue components of PAR (**b**).

Images of individual valves placed on a fused quartz slide have been acquired at different distances along the optical axis when illuminated by radiation of different spectral contents, in order to experimentally verify what we evaluated numerically. In Figure 4 and Figure 5 the results relative to an example case are shown, but consistent outcomes have been obtained for all the examined valves. When illuminated by an halogen lamp (λ = 400–1100 nm), valves appear quite transparent (due to low absorption of silica in visible-near infrared spectral range) and iridescent (because of diffraction induced by the porous ultrastructure), as can be seen in Figure 4a, left side. Starting from a distance of about z=7 μm from the valve, the radiation starts to confine both along the optical axis in a central, bright hotspot and along the apical axis in a less intense light strip, in accordance to numerical simulations (see Figure 2b). At a distance of about z=12 μm from the valve, the ratio of the hotspot intensity ($I_{\mathrm{spot}}$) to the incident intensity ($I_{\mathrm{in}}$) reaches a maximum value of 1.73±0.01 (see Figure 4a, right side). When illuminated by UV-B radiation (λ = 280–315 nm), obtained by properly filtering the emission of a deuterium lamp, the valve looks mainly dark due to absorption by frustule silica (see Figure 4b, left side), while the *fascia* scatters radiation. In general, transmitted intensity is quite lower compared to visible radiation and weakly confined at higher distances from the valve, with a plateau localized between *z* = 12 and *z* = 24 μm. The central hotspot reaches a maximum value at a distance of about z=19 μm from the valve, with a $I_{\mathrm{spot}}/I_{\mathrm{in}}$ ratio of 1.49±0.01 (see Figure 4b, right side).

These results have been summarized in Figure 5a, where the values of the $I_{\mathrm{spot}}/I_{\mathrm{in}}$ ratio for the two considered spectral ranges (VIS-NIR and UV-B, respectively) have been reported as a function of the distance from the valve (*z*). Furthermore, by using optical band-pass filters centered at 640, 532, and 460 nm respectively, we have been able to evaluate analogously the behavior of transmitted light in red, green and blue specral ranges (main components of PAR, see Figure 5b). The results show that, the shorter the wavelength of the incoming radiation, the farther from the valve the hotspot reaches its maximum in intensity. For sufficiently short wavelengths (UV-B), the confinement of radiation along the optical axis is weaker, less localized, and takes place quite far from the valve, mostly outside the frustule (if we take into account the dimension of the whole cell). Plus, the intensity of UV-B incident radiation is noticeably attenuated by diatom biosilica.

**Figure 6:**
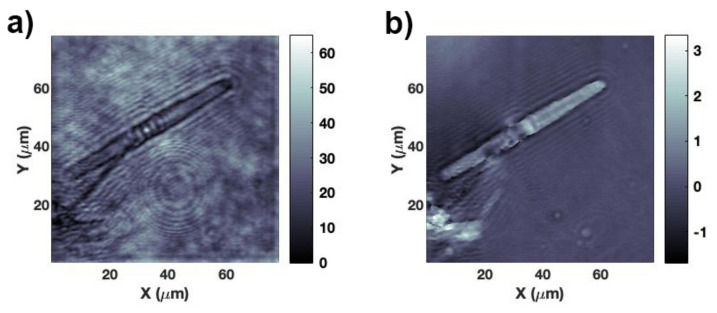
Amplitude (**a**) and phase (**b**) maps reconstructed at a distance z=10.5 μm from a valve irradiated by a laser beam at λ=660 nm. In the phase map, brighter regions correspond to higher values of the optical path length. Amplitude is expressed in arbitrary units while phase in radiants.

In this kind of experiments, the incoming radiation is not perfectly collimated and refraction induced by the supporting quartz slide may in principle deflect the direction of propagation of light. In order to understand if and at which extent the observed light confinement effect is affected by the presence of the supporting silica slide, digital holography characterization has been performed by making use of the interferometric set-up described in Section 2.7.1. Digital holography allows, by a single acquisition, an accurate reconstruction of both the amplitude and the phase of the optical field which interacts with the valve at every position along the optical axis and at high spatial resolution (~λ/20). The hologram of the valve is acquired in z=0 plane, while the silica slide lies in z<0 half-space. Thus, the presence of the silica slide is excluded in the reconstruction of the complex field of the object beam, which in fact is numerically propagated starting from the frequency spectrum of the acquired hologram. In Figure 6 both the amplitude and the phase maps retrieved at a distance of z=10.5 μm from a single valve are shown, for an incoming wavelength of 660 nm. At this distance $I_{\mathrm{spot}}$ reaches its maximum value, in accordance with transmission measurements performed in the case of incoherent red irradiation (see Figure 5b, red curve). The phase map allows the quantification of the retardation undergone by light when propagating through the valve (see Figure 6b). We can finally conclude that light confinement is really induced by valve morphology and refractive index contrast relative to the surrounding environment and not due to any artifacts caused by the presence of the supporting quartz slide nor by aberrations of the collection optics.

### 3.3. Photoluminescence Properties of the Frustule

Nanostructured silica, e.g., in the form of nanoparticles, oxidized porous silicon, sol gels, and silicon-oxide thin films, is characterized by strong photoluminescence emission in the visible spectral range after excitation with UVR [61]. This process is forbidden in bulk, amorphous silica due to its wide band gap (~11 eV) and it is mainly caused by the presence of surface defects (non-bridging oxygen hole centers, neutral oxygen vacancies, silanol groups) and to the recombination of self-trapped excitons [62,63]. Diatom biosilica does not represent an exception, and its photoluminescence characteristics (which are affected by the chemical composition of the environment) have been successfully exploited as transducing mechanism in several sensing and biosensing schemes [27,28,64].

**Figure 7:**
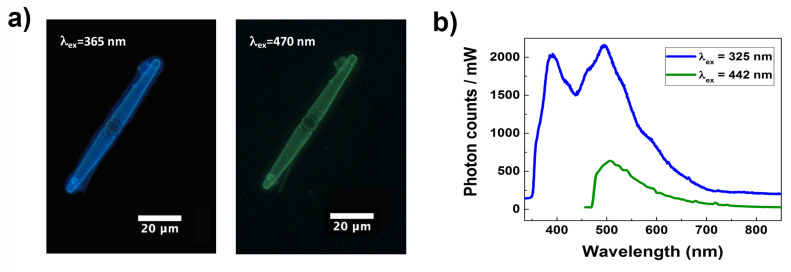
Photoluminescence characterization of a single valve of *C. pulchella* diatom. Fluorescence imaging after excitation with incoherent radiation at 365 (left) and at 470 nm (right) (**a**). Comparison of emission spectra after laser excitation at 325 (blue curve) and 442 nm (green curve) (**b**).

In Figure 7a, images of a single valve of *C. pulchella* acquired by fluorescence microscopy after excitation at 365 nm (UV-A) and 470 nm (blue) are shown, demonstrating that light emission from diatom biosilica can also be triggered by visible radiation, as already observed for other species [36], with an intense cyan-blue emission after UV-A excitation and a less bright green emission after blue excitation, respectively. It can thus be useful to compare the efficiency of these different emission pathways. At this aim we performed steady-state photoluminescence spectroscopy on a dense sample of cleaned diatom valves deposited onto a silicon slide, exciting it at 325 nm (at the boundaries between UV-B and UV-A) and 442 nm (violet-blue) making use of a continuous wave He-Cd laser (see Figure 7b). The spectra have been acquired with the same integration time and with the same incident power, in order to perform a consistent comparison. After excitation at 325 nm, the acquired spectrum presents multiple peaks (i.e., the investigated band is a combination of several transitions), with two main maxima at λ=390 nm (UV-A) and λ=495 nm (cyan). Two less pronounced shoulders are convolved and detectable at about λ=425 nm (violet) and λ=462 nm (blue). It is worthwhile to highlight that in the observed spectral range of emission fall the absorption spectra of many of the pigments commonly present in diatoms and involved in light harvesting and photoprotection (in particular chlorophyll *a*, chlorophyll *c*, fucoxanthin, and diadinoxanthin [65,66]). On the other side, as can be seen looking again at Figure 7b, green emission induced by excitation in violet-blue spectral range looks weaker in the same conditions of acquisition. We can conclude that, compared to other photoluminescence emission patterns, the one caused by UVR excitation results in a more efficient conversion of detrimental radiation in PAR, thus increasing photosynthetic productivity. Together with absorption by biosilica and relocation of transmitted intensity by diffraction, UVR-VIS wavelength conversion mediated by nanostructured silica photoluminescence may represent a further, sophisticated mechanism by which frustule is able to significantly screen the living cell from harmful optical radiation [33,34,35,36].

### 3.4. Interaction of Living Cells with Optical Radiation

Once the behavior of *C. pulchella* valves in the interaction with optical radiation of different spectral content has been analyzed, the next step consisted in the study of their influence on living cells when exposed to both PAR and UVR after diverse irradiation treatments. As in the case of bare frustules, we performed both numerical simulations and experimental characterization by transmission imaging at different spectral ranges.

**Figure 8:**
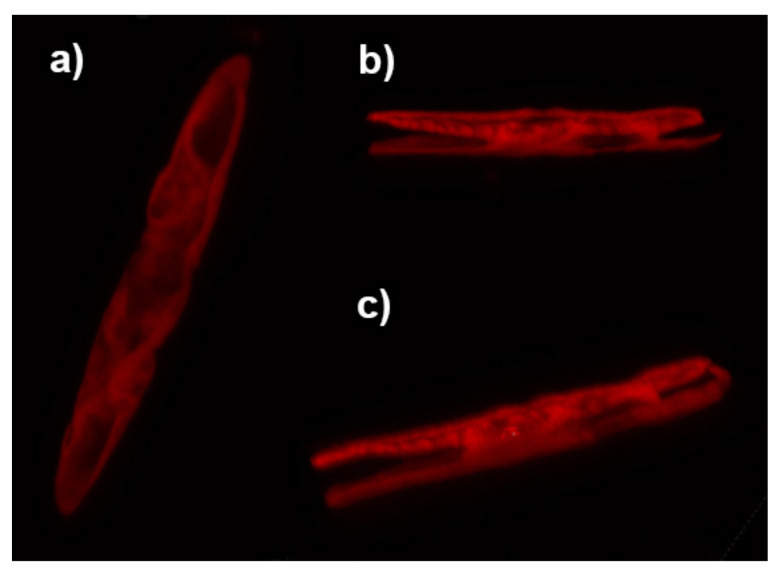
Top (**a**), lateral (**b**), and angled (**c**) view of a partially joined plastid pair inside a living *C. pulchella* cell as reconstructed by CLSM. Red emission peaked at about λ=680 nm is due to plastid autofluorescence following excitation by a diode laser emitting at λ=637.1 nm.

**Figure 9:**
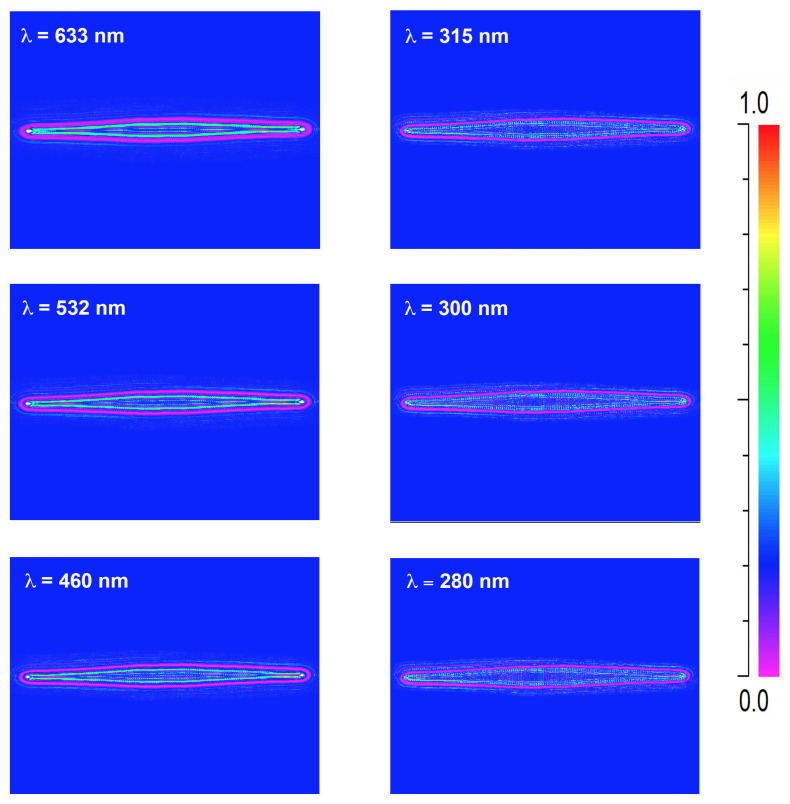
Spatial distribution of field intensity inside a *C. pulchella* single cell when irradiated with optical radiation at different wavelengths. The cell is placed between z=0 and z=10 μm, while the field is numerically evaluated by WA-BPM at z=5 μm. Incident power: 0.2 (a.u.).

Diatoms of the genus *Ctenophora* are generally provided with two, plate-like plastids [7] where the photosynthetic processes take place. They occupy most of the internal volume of the cell, as can be observed from confocal laser scanning microscopy (CLSM) characterization of single individuals (see Figure 8). We thus approximated the entire cell for numerical simulations as a film characterized by a refractive index of n=1.5 (slightly smaller than the typical refractive index of microalgae plastids as reported in Refs. [67,68]) and enclosed by two valves. The cell has been considered immersed in water. Dispersion of silica and water and absorption of silica have been taken into account. Absorption by plastids has been neglected since we are interested in retrieving the spatial distribution of the optical field inside the cell and in understanding its potential matching with the plastid itself. Considering the average height of a *C. pulchella* individual (~10 μm), we evaluated the intensity distribution of the optical field inside the cell (z=5 μm, with the cell extending from z=0 μm to z=10 μm) when irradiated by a plane wave at different wavelengths in UV-VIS spectral range. As can be seen from Figure 9, in case of blue, green, and red radiation propagating inside the cell, a sort of eye-shaped intensity pattern takes place, overlapping to the plastid. Moving towards shorter wavelengths, this pattern progressively reduces and finally almost vanishes in UV-B spectral range. Combining these results with the numerical simulations conducted on bare valves allows inferring that, while visible radiation, after collection and deflection induced by ultrastructure diffraction, efficiently superimposes on the plastids, UV-B radiation is strongly attenuated by frustule silica. The portion of UVR which is not extinguished by silica is confined by the valve well outside the cell (see Figure 3). In general, the refractive index of the different cell organelles varies between approximately 1.36 of cytoplasm up to 1.51 of the plastid and cell wall [69], thus our simulations are based on a very simplified model which does not take into account the complex composition of the interior of the cell and an experimental validation of these findings is mandatory.

**Figure 10:**
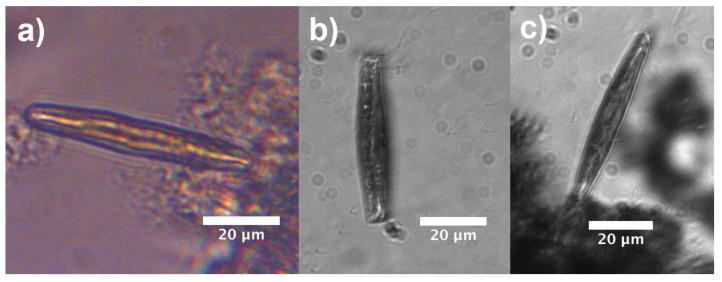
Single, living *C. pulchella* cells immersed in water and illuminated by VIS-NIR radiation (**a**) and UV-B radiation (girdle view in (**b**) and valvar view in (**c**)).

Observations relative to living control cells immersed in water are reported in Figure 10. The cells have been grown under the conditions described in Section 2.1 (irradiation with 40 μmol photons $m^{-2}$ $s^{-1}$ PAR 16 h light: 8 h dark). When illuminated by a halogen lamp (λ= 400–1100 nm), the cell shows a light pattern throughout its internal volume whose shape is similar to that retrieved by numerical simulations, implying that the optical field is efficiently overlapped to the plastids (Figure 10a). No light patterns are detectable in case of UV-B illumination by a filtered deuterium lamp, and in general the cell looks opaque due to absorption by biosilica (see Figure 10b,c). A similar behavior has been observed for cells which have previously undergone a 7 days treatment with 120 μmol photons $m^{-2}$ $s^{-1}$ PAR 16 h light: 8 h dark (see Supplementary Materials).

Analogous analyses have been conducted after different irradiation treatments with UVR, summarized in Table 1 (PA standing for 120 μmol photons $m^{-2}$ $s^{-1}$ PAR + 5 W $m^{-2}$ UV-A 13–16 h light: 11–8 h dark, the PAR lasted 3 h longer; PAB standing for 120 μmol photons $m^{-2}$ $s^{-1}$ PAR + 5 W $m^{-2}$ UV-A + 1–1.4 W $m^{-2}$ UV-B 13–16 h light: 11–8 h dark, the PAR lasted 3 h longer). The parameters relative to the photosynthetic activity and to the growth rate after 7-days treatment are also reported, $F_{V}/F_{m}$ standing for the maximum quantum yield, $rETR_{max}$ for the relative electron transport rate, $I_{k}$ for the light saturation point, α for the photosynthetic efficiency, NPQ for the non-photochemical quenching, and μ fot the specific growth rate. It is worthwhile to notice how the cells still show a positive growth rate, continuing their division processes, even after UVR harsh treatments. Since in the present study specific UV-absorbing substances were not detectable in a significant amount in intact cells (see Supplementary Materials), the observed results are probably ascribable to the screening action carried out by the frustule.

**Figure 11:**
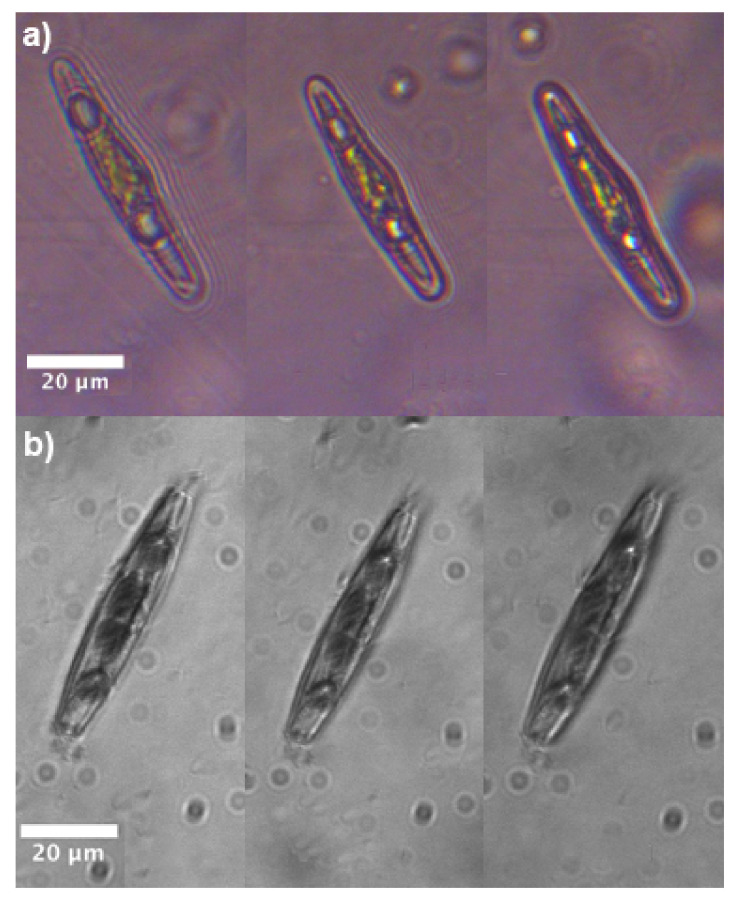
Transmission imaging scans (step: 5 μm) of single living *C. pulchella* cells which underwent PAR + UV-A + UV-B treatment, when illuminated with VIS-NIR (**a**) and UV-B (**b**) radiation, respectively.

Cells which underwent 7-days treatment with UVR are characterized by the presence of typically 2–4 oil droplets (see Figure 11 for PAB-treated cells). Oil droplets (also known as oil bodies, lipid droplets or lipid bodies [70]), mainly consist of triacylglycerols (TAGs), chrysolaminarin, and proteins [71], and accumulate in microalgae in response to various stress conditions (e.g., nutrient starvation, high light exposure, hypoxia, increased salinity, and chemical treatment [72,73,74,75]). Their functions comprise energy storage under adverse conditions, depot of acyl chains for membrane synthesis, and cell signaling [70] and they have recently attracted great attention for their potential use in biofuel production [71]. When *C. pulchella* cells previously treated with PAR + UV-A + UV-B are illuminated with VIS-NIR radiation, light still couples to the plastids (see iridescence inside the cell in Figure 11a) but is also strongly coupled to the oil droplets standing at the polar regions. When irradiated by UV-B (see Figure 11b), scattering by oil droplets is still present even if quite weaker, but no detectable radiation superimposed on the plastids is present inside the cell. Analogous behavior is observable for PA-treated cells (see Supplementary Materials together with holographic characterization of oil droplets). We can conclude that, in stress conditions induced by prolonged and harsh UVR exposure and in general by an excess of incoming light, the shielding action of the frustule combines with the synthesis of lipid bodies, which is typical of most microalgae, besides diatoms.

## 4. Conclusions

The role of the frustule in the extraordinary evolutionary and ecological success of diatoms is probably related to several, different functions, comprising mechanical stability, fluid-dynamics, and sunlight harvesting for photosynthesis. In particular, its interaction with UVR has been the object of several, recent studies which, nevertheless, were focused on the optical characterization of bare frustules rather than on its interplay with the living organisms (and specifically with plastids) in their own aquatic environment [33,34,35,36]. We started from the study of the behavior of pennate, araphide *C. pulchella* diatoms, whose growth seems to be not significantly affected by harsh UVR treatments, to study the role of their frustule in the interaction with optical radiation and its interplay with plastids in living individuals. Numerical simulations, transmission imaging measurements and digital holography allowed the understanding of the mechanisms by which *C. pulchella* valves are able to efficiently collect and confine PAR in multiple directions while absorbing most of the incident UVR and spatially relocating outside the cell the portion which is not extinguished. Furthermore, fluorescence imaging and photoluminescence spectroscopy revealed an efficient conversion of UVR in visible radiation matching the abosption spectra of the main chloroplasts present in diatoms. Finally, the study of the interaction of light of different spectral content with living *C. pulchella* cells showed the formation of standing intensity patterns efficiently ovelapped to plastids in case of visible irradiation, while propagation of UV-B radiation is substantially inhibited inside the diatom protoplasm.

## Figures and Tables

**Table 1:** Photosynthetic activity parameters and specific growth rate for a culture of *C. pulchella* cells after different irradiation treatments. See text for details and parameters definition. Standard deviation is reported between brackets (n=3).

	Photosynthetic Activity					Specific
						Growth Rate
	$F_{V}/F_{m}$	${\mathrm{rETR}}_{\max}$ **(Rel. Units)**	$I_{k}$ (μMol Photons $m^{-2}$ $s^{-1}$)	α%	NPQ	μ (${\mathrm{day}}^{-1}$)
*Start*	0.470 (0.015)	93 (11)	610 (30)	0.15 (0.06)	0.59 (0.08)	
*7 days treatment:*						
**Control**	0.541 (0.019)	100 (10)	610 (30)	0.16 (0.07)	0.56 (0.02)	0.091 (0.005)
**PAR**	0.34 (0.04)	119 (13)	680 (30)	0.17 (0.05)	1.12 (0.15)	0.118 (0.014)
**PA**	0.29 (0.05)	149 (14)	700 (30)	0.21 (0.09)	1.00 (0.08)	0.080 (0.003)
**PAB**	0.28 (0.03)	118 (14)	690 (30)	0.13 (0.06)	1.00 (0.09)	0.085 (0.008)

## Data Availability

The data presented in this study are available on request from the corresponding author.

## Supplementary Materials

The following are available online at https://www.mdpi.com/article/10.3390/nano11112855/s1: Table S1: Low-order Padé approximants expressed in terms of the operator *P*. Figure S1: Images of the inner (**a**) and outer (**b**) sides of a single *C. pulchella* valve as obtained by SEM. Corresponding binary images obtained by proper thresholding (**c**,**d**). Related refractive index maps when air is considered as the surrounding environment (**e**,**f**). Figure S2: Refractive index profile of a single *C. pulchella* valve immersed in air for λ=633 nm, XZ view. The valve extends from z=0 to z=0.4 μm. The red arrow on the left shows the direction of propagation of the incoming field. Figure S3: Refractive index profile of a *C. pulchella* live cell immersed in water for λ=633 nm, XZ view. Figure S4: Two *C. pulchella* live cells immersed in water and irradiated with VIS-NIR light (λ = 400–1100 nm) after PAR treatment, girdle view (**a**). Single live cell immersed in water and irradiated with UVB (λ = 280–315 nm) after PAR treatment, valve view (**b**). Figure S5: Transmission imaging scans (step: 5 μm) of single *C. pulchella* live cells after PAR + UVA treatment, when shined with VIS-NIR (**a**) and UVB (**b**) radiation, respectively. Figure S6: Amplitude and phase maps of a live cell treated with PAR + UVA + UVB radiation. Axis units in microns. Figure S7: Chromatogram of a UV-treated sample (solid line) and control sample (dashed line) of *C. pulchella* revealing no significant absorption contribution around λ=334 nm if compared to absorption by other pigments.

## Abbreviations

The following abbreviations are used in this manuscript:

UVR	Ultra-violet radiation
VIS	Visible radiation
NIR	Near-infrared radiation
PAR	Photosynthetic active radiation
PER	Photo-enzymatic repair photoreactivation
MAA	Mycosporine-like amino acid
HPLC	High performance liquid chromatography
rETR	Relative electron transport rate
PFR	Photon fluence rate
WA-BPM	Wide-angle beam propagation method
CAD	Computer-aided design
FWHM	Full width at half maximum
CMOS	Complementary metal-oxide semiconductor
CCD	Charged-coupled device
SEM	Scanning electron microscopy
CSLM	Confocal laser scanning microscopy

## References

[1] Seckbach J., Kociolek P. (2011). The Diatom World.

[2] Leblanc K., Queguiner B., Diaz F., Cornet V., Michel-Rodriguez M., de Madron X.D., Bowler C., Malviya S., Thyssen M., Grégori G. (2018). Nanoplanktonic diatoms are globally overlooked but play a role in spring blooms and carbon export. Nat. Commun..

[3] Hildebrand M., Lerch S.J., Shrestha R.P. (2018). Understanding diatom cell wall silicification—Moving forward. Front. Mar. Sci..

[4] Nelson D.M., Tréguer P., Brzezinski M.A., Leynaert A., Quéguiner B. (1995). Production and dissolution of biogenic silica in the ocean: Revised global estimates, comparison with regional data and relationship to biogenic sedimentation. Glob. Biogeochem. Cycles.

[5] Smol J.P., Stoermer E.F. (2010). The Diatoms: Applications for the Environmental and Earth Sciences.

[6] Zgłobicka I., Gluch J., Liao Z., Werner S., Guttmann P., Li Q., Bazarnik P., Plocinski T., Witkowski A., Kurzydlowski K.J. (2021). Insight into diatom frustule structures using various imaging techniques. Sci. Rep..

[7] Round F.E., Crawford R.M., Mann D.G. (1990). Diatoms: Biology and Morphology of the Genera.

[8] De Tommasi E., Gielis J., Rogato A. (2017). Diatom frustule morphogenesis and function: A multidisciplinary survey. Mar. Genom..

[9] Losic D., Mitchell J.G., Lal R., Voelcker N.H. (2007). Rapid fabrication of micro-and nanoscale patterns by replica molding from diatom biosilica. Adv. Funct. Mater..

[10] Hamm C.E., Merkel R., Springer O., Jurkojc P., Maier C., Prechtel K., Smetacek V. (2003). Architecture and material properties of diatom shells provide effective mechanical protection. Nature.

[11] Hale M.S., Mitchell J.G. (2001). Functional morphology of diatom frustule microstructures: Hydrodynamic control of Brownian particle diffusion and advection. Aquat. Microb. Ecol..

[12] Hale M.S., Mitchell J.G. (2002). Effects of particle size, flow velocity, and cell surface microtopography on the motion of submicrometer particles over diatoms. Nano Lett..

[13] Waite A., Fisher A., Thompson P.A., Harrison P.J. (1997). Sinking rate versus cell volume relationships illuminate sinking rate control mechanisms in marine diatoms. Mar. Ecol. Prog. Ser..

[14] De Tommasi E. (2016). Light manipulation by single cells: The case of diatoms. J. Spectrosc..

[15] De Stefano L., Rea I., Rendina I., De Stefano M., Moretti L. (2007). Lensless light focusing with the centric marine diatom Coscinodiscus walesii. Opt. Express.

[16] De Tommasi E., Rea I., Mocella V., Moretti L., De Stefano M., Rendina I., De Stefano L. (2010). Multi-wavelength study of light transmitted through a single marine centric diatom. Opt. Express.

[17] Ferrara M.A., Dardano P., De Stefano L., Rea I., Coppola G., Rendina I., Congestri R., Antonucci A., De Stefano M., De Tommasi E. (2014). Optical properties of diatom nanostructured biosilica in Arachnoidiscus sp: Micro-optics from mother nature. PLoS ONE.

[18] Fuhrmann T., Landwehr S., El Rharbi-Kucki M., Sumper M. (2004). Diatoms as living photonic crystals. Appl. Phys. B.

[19] Goessling J.W., Wardley W.P., Lopez-Garcia M. (2020). Highly Reproducible, Bio-Based Slab Photonic Crystals Grown by Diatoms. Adv. Sci..

[20] Rogato A., De Tommasi E. (2020). Physical, Chemical, and Genetic Techniques for Diatom Frustule Modification: Applications in Nanotechnology. Appl. Sci..

[21] Bhardwaj V., Mirliss M.J. (2001). Diatomaceous Earth filtration for drinking water. National Drinking Water Clearing House Fact Sheet.

[22] Verma K. (2013). Role of diatoms in the world of forensic science. J. Forensic Res..

[23] De Tommasi E., De Luca A., Lavanga L., Dardano P., De Stefano M., De Stefano L., Langella C., Rendina I., Dholakia K., Mazilu M. (2014). Biologically enabled sub-diffractive focusing. Opt. Express.

[24] Fang Y., Chen V.W., Cai Y., Berrigan J.D., Marder S.R., Perry J.W., Sandhage K.H. (2012). Biologically enabled syntheses of freestanding metallic structures possessing subwavelength pore arrays for extraordinary (surface plasmon-mediated) infrared transmission. Adv. Funct. Mater..

[25] Managò S., Zito G., Rogato A., Casalino M., Esposito E., De Luca A.C., De Tommasi E. (2018). Bioderived three-dimensional hierarchical nanostructures as efficient surface-enhanced raman scattering substrates for cell membrane probing. ACS Appl. Mater. Interfaces.

[26] Kumari S., Min K.H., Kanth B.K., Jang E.K., Pack S.P. (2020). Production of TiO_2_-deposited Diatoms and Their Applications for Photo-catalytic Degradation of Aqueous Pollutants. Biotechnol. Bioprocess Eng..

[27] De Stefano L., Rotiroti L., De Stefano M., Lamberti A., Lettieri S., Setaro A., Maddalena P. (2009). Marine diatoms as optical biosensors. Biosens. Bioelectron..

[28] Gale D.K., Gutu T., Jiao J., Chang C.H., Rorrer G.L. (2009). Photoluminescence detection of biomolecules by antibody-functionalized diatom biosilica. Adv. Funct. Mater..

[29] Toster J., Iyer K.S., Xiang W., Rosei F., Spiccia L., Raston C.L. (2013). Diatom frustules as light traps enhance DSSC efficiency. Nanoscale.

[30] Bandara T., Furlani M., Albinsson I., Wulff A., Mellander B.E. (2020). Diatom frustules enhancing the efficiency of gel polymer electrolyte based dye-sensitized solar cells with multilayer photoelectrodes. Nanoscale Adv..

[31] Rea I., Martucci N.M., De Stefano L., Ruggiero I., Terracciano M., Dardano P., Migliaccio N., Arcari P., Taté R., Rendina I. (2014). Diatomite biosilica nanocarriers for siRNA transport inside cancer cells. Biochim. Biophys. Acta (BBA)—Gen. Subj..

[32] Managò S., Tramontano C., Delle Cave D., Chianese G., Zito G., De Stefano L., Terracciano M., Lonardo E., De Luca A.C., Rea I. (2021). SERS Quantification of Galunisertib Delivery in Colorectal Cancer Cells by Plasmonic-Assisted Diatomite Nanoparticles. Small.

[33] Ellegaard M., Lenau T., Lundholm N., Maibohm C., Friis S.M.M., Rottwitt K., Su Y. (2016). The fascinating diatom frustule—Can it play a role for attenuation of UV radiation?. J. Appl. Phycol..

[34] Su Y., Lenau T.A., Gundersen E., Kirkensgaard J.J., Maibohm C., Pinti J., Ellegaard M. (2018). The UV filtering potential of drop-casted layers of frustules of three diatom species. Sci. Rep..

[35] Aguirre L.E., Ouyang L., Elfwing A., Hedblom M., Wulff A., Inganäs O. (2018). Diatom frustules protect DNA from ultraviolet light. Sci. Rep..

[36] De Tommasi E., Congestri R., Dardano P., De Luca A.C., Managò S., Rea I., De Stefano M. (2018). UV-shielding and wavelength conversion by centric diatom nanopatterned frustules. Sci. Rep..

[37] Karentz D., Cleaver J.E., Mitchell D.L. (1991). Cell Survival Characteristics and Molecular Responses of Antarctic Phytoplankton to Ultraviolet-B Radiation 1. J. Phycol..

[38] Smith R.C., Prezelin B., Baker K., Bidigare R., Boucher N., Coley T., Karentz D., MacIntyre S., Matlick H., Menzies D. (1992). Ozone depletion: Ultraviolet radiation and phytoplankton biology in Antarctic waters. Science.

[39] Kiefer J. (2012). Biological Radiation Effects.

[40] Jeffrey S., MacTavish H., Dunlap W., Vesk M., Groenewoud K. (1999). Occurrence of UVA-and UVB-absorbing compounds in 152 species (206 strains) of marine microalgae. Mar. Ecol. Prog. Ser..

[41] Xiong F., Komenda J., Kopeckỳ J., Nedbal L. (1997). Strategies of ultraviolet-B protection in microscopic algae. Physiol. Plant..

[42] Wulff A., Nilsson C., Sundbäck K., Odmark S.Å.W.S. (1999). UV radiation effects on microbenthos—A four month field experiment. Aquat. Microb. Ecol..

[43] Wulff A., Roleda M.Y., Zacher K., Wiencke C. (2008). UV radiation effects on pigments, photosynthetic efficiency and DNA of an Antarctic marine benthic diatom community. Aquat. Biol..

[44] Wulff A., Zacher K., Hanelt D., Al-Handal A., Wiencke C. (2008). UV radiation—A threat to Antarctic benthic marine diatoms?. Antarct. Sci..

[45] Wulff A., Wängberg S.Å.k., Sundbäck K., Nilsson C., Underwood G.J. (2000). Effects of UVB radiation on a marine microphytobenthic community growing on a sand-substratum under different nutrient conditions. Limnol. Oceanogr..

[46] Zacher K., Hanelt D., Wiencke C., Wulff A. (2007). Grazing and UV radiation effects on an Antarctic intertidal microalgal assemblage: A long-term field study. Polar Biol..

[47] Sundbäck K., Nilsson C., Odmark S., Wulff A. (1996). Does ambient UV-B radiation influence marine diatom-dominated microbial mats? A case study. Aquat. Microb. Ecol..

[48] Underwood G.J., Nilsson C., Sundbäck K., Wulff A. (1999). Short-term effects of UVB radiation on chlorophyll fluorescence, biomass, pigments, and carbohydrate fractions in a benthic diatom mat. J. Phycol..

[49] Sliney D.H. (2007). Radiometric quantities and units used in photobiology and photochemistry: Recommendations of the Commission Internationale de l’Eclairage (International Commission on Illumination). Photochem. Photobiol..

[50] Baker N.R. (2008). Chlorophyll fluorescence: A probe of photosynthesis in vivo. Annu. Rev. Plant Biol..

[51] Helbling E.W., Villafane V.E., Buma A.G., Andrade M., Zaratti F. (2001). DNA damage and photosynthetic inhibition induced by solar ultraviolet radiation in tropical phytoplankton (Lake Titicaca, Bolivia). Eur. J. Phycol..

[52] Vass I., Szilárd A., Sicora C. (2016). Adverse effects of UV-B light on the structure and function of the photosynthetic apparatus. Section XIII: Photosynthesis under environmental stress conditions. Handbook of Photosynthesis.

[53] Hadley G.R. (1992). Wide-angle beam propagation using Padé approximant operators. Opt. Lett..

[54] Hadley G.R. (1992). Multistep method for wide-angle beam propagation. Opt. Lett..

[55] Yu L., Cai L. (2001). Iterative algorithm with a constraint condition for numerical reconstruction of a three-dimensional object from its hologram. J. Opt. Soc. Am. A.

[56] Goodman J.W. (1996). Introduction to Fourier Optics.

[57] Nazarathy M., Shamir J. (1980). Fourier optics described by operator algebra. J. Opt. Soc. Am..

[58] Born M., Wolf E. (2013). Principles of Optics: Electromagnetic Theory of Propagation, Interference and Diffraction of Light.

[59] Rodríguez-de Marcos L.V., Larruquert J.I., Méndez J.A., Aznárez J.A. (2016). Self-consistent optical constants of SiO_2_ and Ta_2_O_5_ films. Opt. Mater. Express.

[60] Hale G.M., Querry M.R. (1973). Optical constants of water in the 200-nm to 200-μm wavelength region. Appl. Opt..

[61] Qin T., Gutu T., Jiao J., Chang C.H., Rorrer G.L. (2008). Photoluminescence of silica nanostructures from bioreactor culture of marine diatom Nitzschia frustulum. J. Nanosci. Nanotechnol..

[62] Itoh C., Tanimura K., Itoh N. (1988). Optical studies of self-trapped excitons in SiO_2_. J. Phys. C Solid State Phys..

[63] Sprynskyy M., Pomastowski P., Hornowska M., Król A., Rafińska K., Buszewski B. (2017). Naturally organic functionalized 3D biosilica from diatom microalgae. Mater. Des..

[64] De Stefano L., Rendina I., De Stefano M., Bismuto A., Maddalena P. (2005). Marine diatoms as optical chemical sensors. Appl. Phys. Lett..

[65] Kuczynska P., Jemiola-Rzeminska M., Strzalka K. (2015). Photosynthetic pigments in diatoms. Mar. Drugs.

[66] Mulders K.J., Lamers P.P., Martens D.E., Wijffels R.H. (2014). Phototrophic pigment production with microalgae: Biological constraints and opportunities. J. Phycol..

[67] Bhowmik A., Pilon L. (2016). Can spherical eukaryotic microalgae cells be treated as optically homogeneous?. J. Opt. Soc. Am. A.

[68] Dauchet J., Blanco S., Cornet J.F., Fournier R. (2015). Calculation of the radiative properties of photosynthetic microorganisms. J. Quant. Spectrosc. Radiat. Transf..

[69] Lehmuskero A., Chauton M.S., Boström T. (2018). Light and photosynthetic microalgae: A review of cellular-and molecular-scale optical processes. Prog. Oceanogr..

[70] Goold H., Beisson F., Peltier G., Li-Beisson Y. (2015). Microalgal lipid droplets: Composition, diversity, biogenesis and functions. Plant Cell Rep..

[71] Maeda Y., Nojima D., Yoshino T., Tanaka T. (2017). Structure and properties of oil bodies in diatoms. Philos. Trans. R. Soc. B Biol. Sci..

[72] Wang Z.T., Ullrich N., Joo S., Waffenschmidt S., Goodenough U. (2009). Algal lipid bodies: Stress induction, purification, and biochemical characterization in wild-type and starchless Chlamydomonas reinhardtii. Eukaryot. Cell.

[73] Siaut M., Cuine S., Cagnon C., Fessler B., Nguyen M., Carrier P., Beyly A., Beisson F., Triantaphylides C., Li-Beisson Y. (2011). Oil accumulation in the model green alga Chlamydomonas reinhardtii: Characterization, variability between common laboratory strains and relationship with starch reserves. BMC Biotechnol..

[74] Boyle N.R., Page M.D., Liu B., Blaby I.K., Casero D., Kropat J., Cokus S.J., Hong-Hermesdorf A., Shaw J., Karpowicz S.J. (2012). Three acyltransferases and nitrogen-responsive regulator are implicated in nitrogen starvation-induced triacylglycerol accumulation in Chlamydomonas. J. Biol. Chem..

[75] Hemschemeier A., Casero D., Liu B., Benning C., Pellegrini M., Happe T., Merchant S.S. (2013). Copper response regulator1—Dependent and—Independent responses of the Chlamydomonas reinhardtii transcriptome to dark anoxia. Plant Cell.

