# Supplementary material for: Underwater Light Manipulation by the Benthic Diatom Ctenophora pulchella: From PAR Efficient Collection to UVR Screening
Source: Nanomaterials (Basel). 2021 Oct 26;11(11):2855. doi: 10.3390/nano11112855 (PMC8621762; doi:10.3390/nano11112855)
Supplement: Supplementary file 1 [file nanomaterials-11-02855-s001.zip › Cteno_SUPPLEMENTARY_MATERIAL final v from authors.pdf]

Underwater light manipulation by the benthic diatom *Ctenophora pulchella*: from PAR efficient collection to UVR screening. Supplementary Material.

Edoardo De Tommasi[1], Ilaria Rea[1], Maria Antonietta Ferrara[1], Luca De Stefano[1], Mario De Stefano[2], Adil. Y. Al-Handal[3], Marija Stamenković[3,4], and Angela Wulff[3]

[1]National Research Council, Institute of Applied Sciences and Intelligent Systems "E. Caianiello", Via P. Castellino 111, I-80131, Naples, Italy
[2]University of Campania "Luigi Vanvitelli", Department of Environmental, Biological, and Pharmaceutical Sciences and Technologies, Via Vivaldi 43, I-81100, Caserta, Italy
[3]University of Gothenburg, Department of Biological and Environmental Sciences, Box 461, 405 30 Göteborg, Sweden
[4]University of Belgrade, Department of Ecology, Institute for Biological Research "Sinisa Stankovic", Bulevar despota Stefana 142, 11060 Belgrade, Serbia

1 Wide angle beam propagation method (WA-BPM)

Beam propagation method (BPM) is usually employed in the study of light propagation in waveguides and optical fibers, i.e. in conditions of paraxiality (small angles respect to the optical axis) and of uniformity of refractive index along the direction of propagation of the field. This is not the case of diatom frustules, where the ultrastructure induces diffraction of light (leading to non-paraxial conditions) and where an abrupt variation in the refractive index between the valve and the surrounding environment takes place.

Starting from the Helmoltz equation:

$$\nabla^2 E(\mathbf{r}) + k^2(\mathbf{r}) = 0 \tag{1}$$

with E electric field, $k = nk_0$ wavenumber (with $k_0 = \frac{2\pi}{\lambda}$ wavenumber in free space), and $n = n(x, y, z)$ refractive index spatial distribution, we can write the solution as:

$$E(\mathbf{r}) = E(x, y, z) = U(x, y, z)e^{-ik_r z} \tag{2}$$

The electric field can thus be expressed as the product of a slowly varying envelope factor $U(x, y, z)$ and a rapid varying phase factor $e^{-ik_r z}$, with $k_r = n_r k_0$ reference wavenumber (expressed in terms of the reference refractive index n_r), which takes into account the average phase variation of the field. We are assuming that the considered wave propagates primarily along z (i.e. we are considering, at first, paraxial conditions). We will also suppose, for now, that the amplitude varies slowly along z axis too. Inserting $U(x, y, z)e^{-ik_r z}$ into Eq. 1 we obtain:

$$\frac{\partial^2 U}{\partial z^2} + 2ik_r \frac{\partial U}{\partial z} + \frac{\partial^2 U}{\partial x^2} + \frac{\partial^2 U}{\partial y^2} + (k^2 - k_r^2)U = 0 \tag{3}$$

Making use of the *slowly varying envelope approximation*:

$$\left| \frac{\partial^2 U}{\partial z^2} \right| \ll \left| 2k_r \frac{\partial U}{\partial z} \right| \tag{4}$$

we obtain the basic BPM equation:

$$\frac{\partial U}{\partial z} = \frac{i}{2k_r} \Big[\frac{\partial^2 U}{\partial x^2} + \frac{\partial^2 U}{\partial y^2} + (k^2 - k_r^2)U \Big] \tag{5}$$

Specifying $U(x, y, z)$ at a plane $z = z_0$, we can iterate U along the z-axis using finite differences for the x and y derivatives.

The most popular BPM variant which can take into account non-paraxial conditions (Wide-Angle Beam Propagation Method, WA-BPM), is known as the *multistep Padé-based technique* . We can denote $\frac{\partial}{\partial z}$ with D, and, consequently, $\frac{\partial^2}{\partial z^2}$ with D^2. Eq. 3 can be now viewed as a quadratic equation to be solved for the differential operator D. This yields to the following solution for a first order equation in z:

$$\frac{\partial U}{\partial z} = ik_r(\sqrt{1 + P} - 1)U \tag{6}$$

with:

$$P \equiv \frac{1}{k_r^2} \Big(\frac{\partial^2}{\partial x^2} + \frac{\partial^2}{\partial y^2} + (k^2 - k_r^2) \Big) \tag{7}$$

Even though it is restricted to forward propagation of the field ($z > 0$), the above equation is exact in that no paraxiality approximation has been introduced. The radical in Eq. 6 can be evaluated by using a Taylor expansion. The first order of the expansion leads to the standard, paraxial BPM, while higher orders lead to more accurate representations of the propagating field. However, expansion

**Padé order (m,n)**	$\mathbf{N_m}$	$\mathbf{D_n}$
(1,0)	$\frac{P}{2}$	1
(1,1)	$\frac{P}{2}$	$1+\frac{P}{4}$
(2,2)	$\frac{P}{2}+\frac{P^2}{4}$	$1+\frac{3P}{4}+\frac{P^2}{16}$
(3,3)	$\frac{P}{2}+\frac{P^2}{2}+\frac{3P^3}{32}$	$1+\frac{5P}{4}+\frac{3P^2}{8}+\frac{P^3}{64}$

Table S1: Low-order Padé approximants expressed in terms of the operator P defined in Eq.7.

via Padé approximants is more accurate than Taylor expansion for the same order of terms. This approach leads to the following equation:

$$\frac{\partial U}{\partial z} = ik\frac{N_m(P)}{D_n(P)}U \tag{8}$$

where N_m and D_n are polynomials in the operator P, and (m, n) is the order of approximation. Some of their low-order values are reported in Table S1. Our numerical capabilities allowed us to make use of the $(1, 1)$ Padé order, corresponding to $N_m = P/2$ and $D_n = 1 + P/4$.

2 Valve CAD retrieval

Three-dimensional CAD models of single valves have been retrieved starting from top view SEM images of the inner and outer side of the valve itself (see Figure S1a and b). The gray-scale SEM images have been properly thresholded and transformed into binary images (see black and white profiles in Figure S1c and d). This allows assigning specific values of refractive index and extinction coefficient to the white (silica) and black (surrounding environment) areas. The obtained valve refractive index maps (see Figure S1e and f) can then be extruded and superimposed in order to obtain a three-dimensional CAD (see Figure S2), while the surrounding environment can be extended through all the volume of interest.

In case of live cells (see Figure S3), a multilayer composed by an inner region with refractive index $n = 1.5$ (slightly smaller than the refractive index of the plastids, $n_p \simeq 1.51$, which occupy most of the inner volume of the cell) in between two valves has been designed, considering water as surrounding medium.

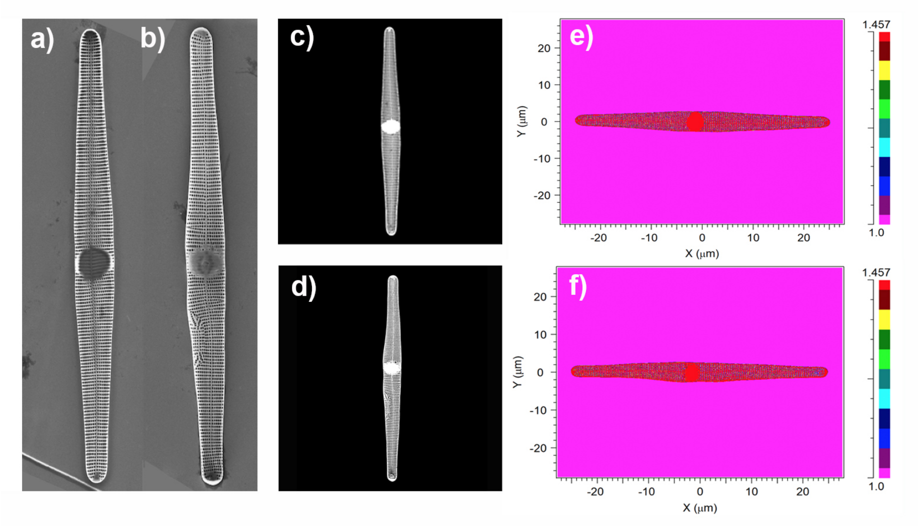

Figure S1: Images of the inner (**a**) and outer (**b**) sides of a single *C. pulchella* valve as obtained by SEM. Corresponding binary images obtained by proper thresholding (**c,d**). Related refractive index maps when air is considered as the surrounding environment (**e,f**).

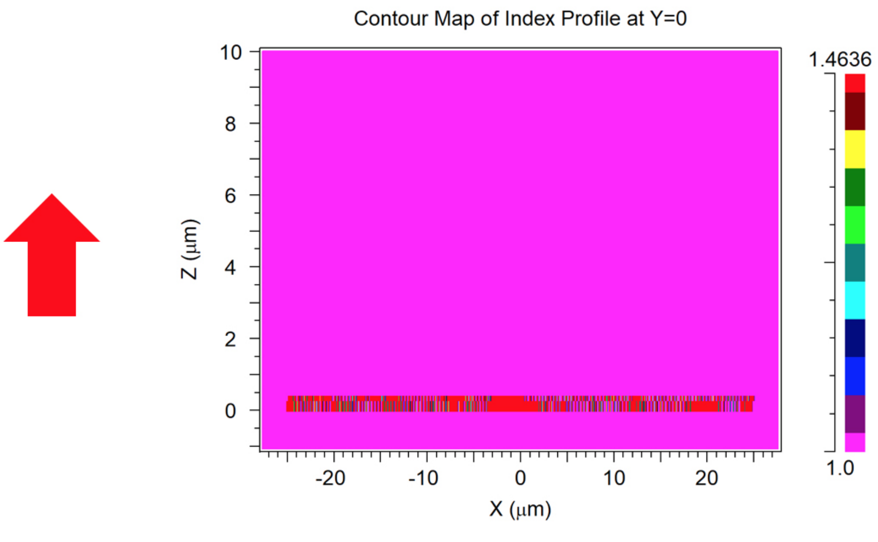

Figure S2: Refractive index profile of a single *C. pulchella* valve immersed in air for $\lambda = 633$ nm, XZ view. The valve extends from $z = 0$ to $z = 0.4$ μm. The red arrow on the left shows the direction of propagation of the incoming field.

3 Live cells treated with PAR

Live *C. pulchella* cells which underwent an intense PAR treatment (7 days irradiation with 120 μmol photons m^{-2} s^{-1} PAR 16 h light : 8 h dark) show a behavior similar to control cells (irradiation with 40 μmol photons m^{-2} s^{-1} PAR 16 h light : 8 h dark) when considering light interaction in different spectral ranges (see Figure S4). VIS-NIR light is efficiently confined inside the protoplasm (see Figure S4a) while no light patterns inside the cell are detectable in case of UVB irradiation (see Figure S4b).

4 Live cells treated with PAR + UVA

Cells which underwent 7 days PAR + UVA treatment (120 μmol photons m^{-2} s^{-1} PAR + 5 W m^{-2} UVA 13-16 h light : 11-8 h dark, the PAR lasted 3 h longer) present a similar behavior respect to cells treated with PAR + UVA + UVB (120 μmol photons m^{-2} s^{-1} PAR + 5 W m^{-2} UVA + 1-1.4 W m^{-2} UVB 13-16 h light: 11-8 h dark, the PAR lasted 3 h longer). In particular, also in this case the formation of oil droplets in response to stress conditions is observed (see Figure S5). Both VIS-NIR and UVB radiation is scattered by the droplets, but only visible radiation is coupled to plastids inducing iridescence while the protoplasm looks dark due to biosilica absorption in case of UVB irradiation.

5 Oil droplets characterization by digital holography

Digital holography performed at $\lambda = 660$ nm on live cells which underwent PAR + UVA + UVB treatment confirm the presence of oil droplets in response to stress conditions. In particular, the bright granular regions detectable in the phase map (see Figure S6) allows stating that lipid bodies are characterized by an higher optical path length respect to the surrounding environment.

6 MAAs detection

Cells were analyzed by spectrophotometric chromatography in order to detect the possible presence of the MMAs usually found in diatoms, i.e. porphyra-334 and shinorine, both characterized by an absorption maximum centered at $\lambda = 334$ nm. Due to their absorption features, it is worth noticing that they are not able to efficiently protect the cell from UV-B ($\lambda = 280 - 315$ nm).

The cells were gently filtered onto a GF/F filter and the filter was put in a mixture of acetone:methanol 80:20. The extract was put in -20 C° for 24 h followed by 1 min ultrasonication using a 3 mm probe (Vibra-cell). The filtrate was purified using a 0.7 mm syringe filter and the solution was analysed

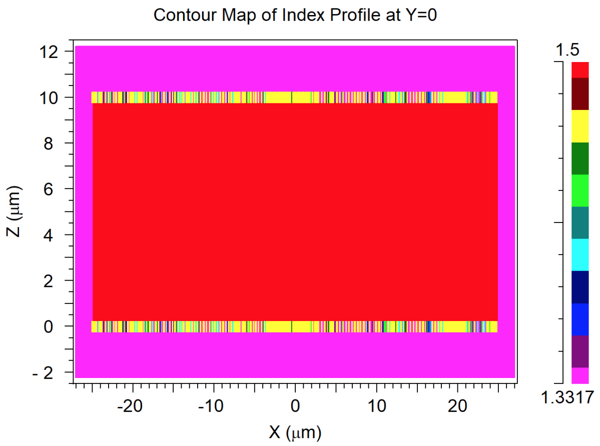

Figure S3: Refractive index profile of a *C. pulchella* live cell immersed in water for $\lambda = 633$ nm, XZ view.

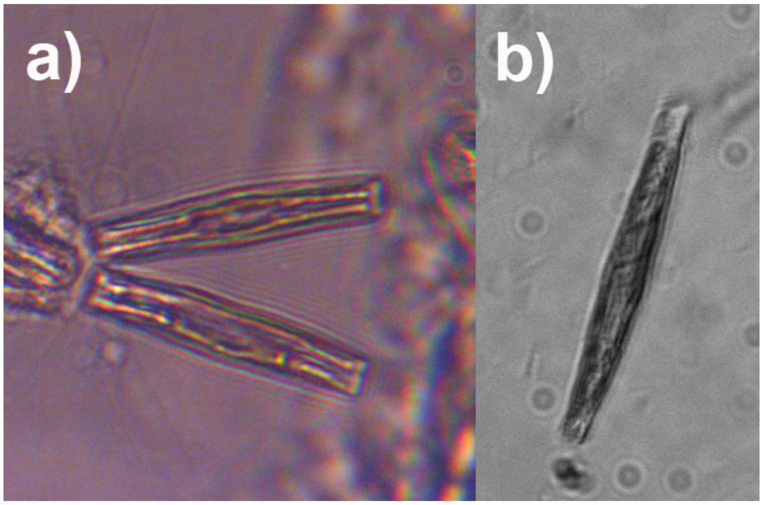

Figure S4: Two *C. pulchella* live cells immersed in water and irradiated with VIS-NIR light ($\lambda = 400 - 1100$ nm) after PAR treatment, girdle view (**a**). Single live cell immersed in water and irradiated with UVB ($\lambda = 280 - 315$ nm) after PAR treatment, valve view (**b**).

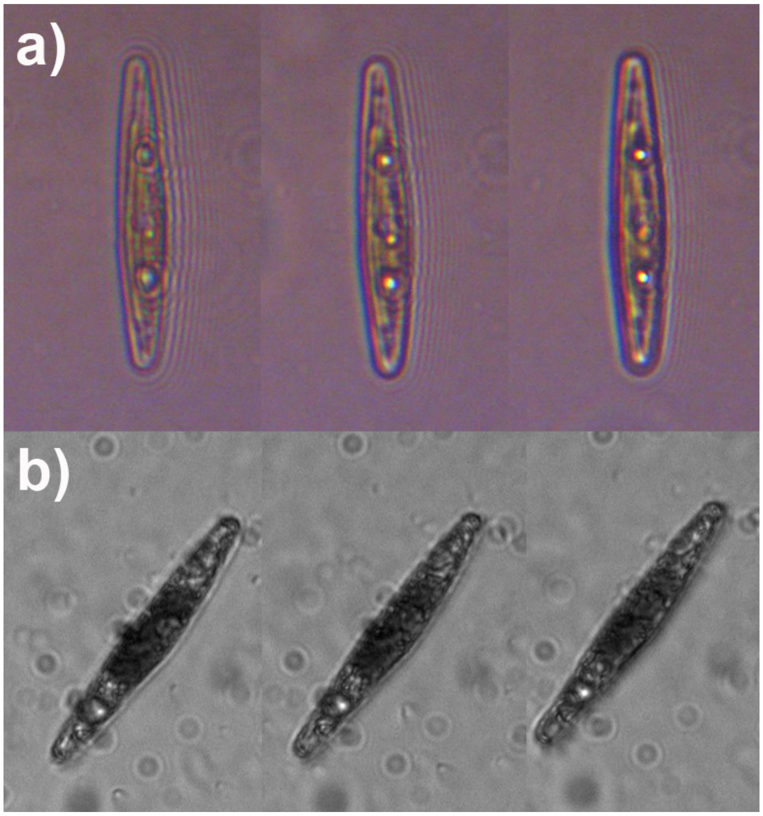

Figure S5: Transmission imaging scans (step: 5 μm) of single *C. pulchella* live cells after PAR + UVA treatment, when shined with VIS-NIR (**a**) and UVB **b**) radiation, respectively.

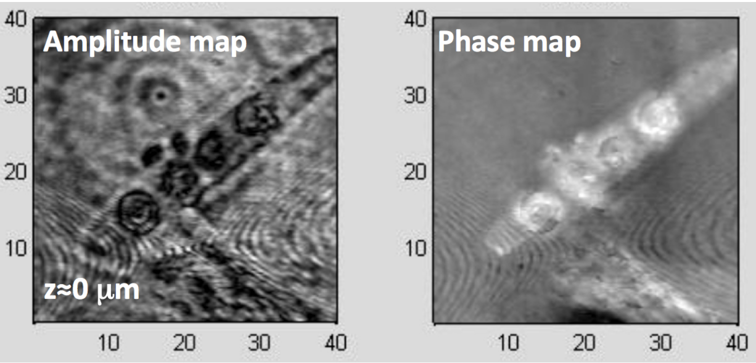

Figure S6: Amplitude and phase maps of a live cell treated with PAR + UVA + UVB radiation. Axis units in microns.

spectrophotometrically between 260-700 nm (see FigureS7). No significant contribution to the signal is detected around $\lambda = 334$ nm if compared to absorption by other pigments.

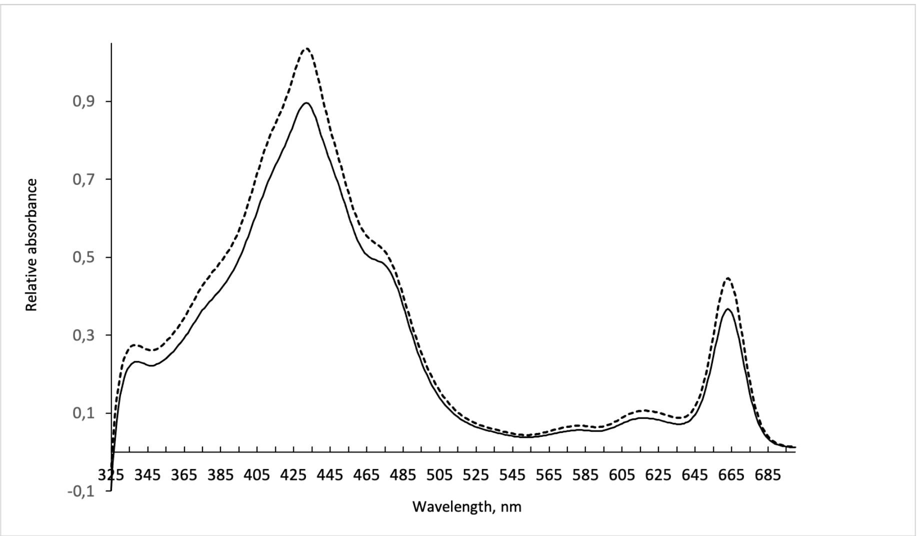

Figure S7: Chromatogram of a UV-treated sample (solid line) and control sample (dashed line) of *C. pulchella* revealing no significant absorption contribution around $\lambda = 334$ nm if compared to absorption by other pigments.
